# RAB27A promotes the proliferation and invasion of colorectal cancer cells

**DOI:** 10.1038/s41598-022-23696-7

**Published:** 2022-11-12

**Authors:** Qingyan Li, Huixia Zhao, Weiwei Dong, Na Guan, Yanyan Hu, Zhiyan Zeng, He Zhang, Fengyun Zhang, Qiuwen Li, Jingwen Yang, Wenhua Xiao

**Affiliations:** 1 Graduate School of Jinzhou Medical University, Liaoning, 121001 China; 2 Senior Department of Oncology, the Fifth Medical Center of PLA General Hospital, Beijing, 100071 China; 3 Department of Oncology, 4th Medical Center of PLA General Hospital, Beijing, 100048 China; 4 Department of Oncology, Suining Central Hospital, Sichuan, 629300 China

**Keywords:** Cancer, Cell biology, Oncology

## Abstract

Colorectal cancer (CRC) is one of the most commonly diagnosed cancer types worldwide. Despite significant advances in prevention and diagnosis, CRC is still one of the leading causes of cancer-related mortality globally. RAB27A, the member of RAB27 family of small GTPases, is the critical protein for intracellular secretion and has been reported to promote tumor progression. However, it is controversial for the role of RAB27A in CRC progression, so we explored the exact function of RAB27A in CRC development in this study. Based on the stable colon cancer cell lines of RAB27A knockdown and ectopic expression, we found that RAB27A knockdown inhibited proliferation and clone formation of SW480 colon cancer cells, whereas ectopic expression of RAB27A in RKO colon cancer cells facilitated cell proliferation and clone formation, indicating that RAB27A is critical for colon cancer cell growth. In addition, our data demonstrated that the migration and invasion of colon cancer cells were suppressed by RAB27A knockdown, but promoted by RAB27A ectopic expression. Therefore, RAB27A is identified as an onco-protein in mediating CRC development, which may be a valuable prognostic indicator and potential therapeutic target for CRC.

## Introduction

Colorectal cancer (CRC) is the second leading cause of cancer-related mortality and the fourth most frequently diagnosed cancer worldwide, accounting for 10.2% of all the new cancer cases, and 9.2% of all cancer deaths^1,2^. It has been reported that men are more susceptible than women to the disease, and most of these cases occur in individuals above 65 years of age^3,4^. Though different risk factors were found to be associated with CRC development, more than two thirds of CRC arise from environmental and genetic factors. Poor dietary habits, alcohol and tobacco consumption, red meat consumption, age, obesity and lack of physical activity are found to play a key role in the pathogenesis of CRC^5,6^. Despite significant advances in prevention and diagnosis, CRC is still one of the most deadly cancers worldwide^7,8^. CRC arises when colonic epithelial cells acquire a series of genetic or epigenetic mutations that increase cell growth and survival^9–11^. Therefore, identification of new genes associated with CRC development is important for early diagnosis and treatment.

Rab proteins are a family of small monomeric Ras-like GTPases, which is composed of more than 70 mammalian members^12^. Rab proteins including RAB10, RAB5A, RAB13 and RAB25 are reported to act as onco-proteins in cancer development. It is reported that knockdown of RAB10 arrests the growth cycle and colony formation, as well as promotes cell apoptosis in hepatocellular carcinoma cells; in addition, high RAB10 expression correlates with poor prognosis of the patients^13^. And over-expression of RAB5A promotes filopodia formation and migration in pancreatic cancer cells, while down-regulation of RAB5A plays an opposite role^14^. Besides, deletion of RAB13 inhibits gastric cancer cell proliferation and promotes apoptosis^15^. And Jeong demonstrates that RAB25 over-expression exacerbates the metastasis and invasion of ovarian cancer cells^16^. Rab27 sub-family belongs to Rab protein family, which consists of two isoforms, including RAB27A and RAB27B^17^. As essential proteins for vesicle exocytosis and exosome release, which are critical for regulating the tumor microenvironment, Rab27 proteins have also been reported to be involved in regulating cancer development^18,19^. It has been reported that RAB27A facilitates breast cancer progression by promoting cancer cell invasion and metastasis^20^. In melanoma, RAB27A has been identified to promote tumor growth by increasing vesicular trafficking and exosome secretion^18,21,22^. In addition, the expression level of RAB27A is positively correlated with grade progression and worse prognosis in all grades of gliomas^23^. These studies demonstrate that RAB27A acts as an onco-protein in promoting human cancer development. However, it is still controversial for the role of RAB27A in mediating CRC progression. It has been reported that RAB27A acts in the downstream of NF-κB signaling pathway to promote the stemness of colon cancer cells via up-regulation of cytokine secretion^24^. However, it is also reported that RAB27A is down-regulated in CRC primary tumor tissues compared with matched adjacent tissues, and down-regulation of RAB27A is associated with advanced TNM stage, distant metastasis, and local recurrence^25^. Therefore, it is necessary to explore the exact role of RAB27A in CRC development.

In this study, stable colon cancer cell lines of RAB27A knockdown and ectopic expression were established. And we found that cell proliferation, clone formation, migration and invasion were suppressed by RAB27A knockdown in SW480 colon cancer cells, but promoted by RAB27A ectopic expression in RKO colon cancer cells. Therefore, our results demonstrate that RAB27A acts as an onco-protein and plays a key role in promoting CRC development.

## Results

### Establishment of RAB27A knockdown and ectopic expression cell lines

**Figure 1:**
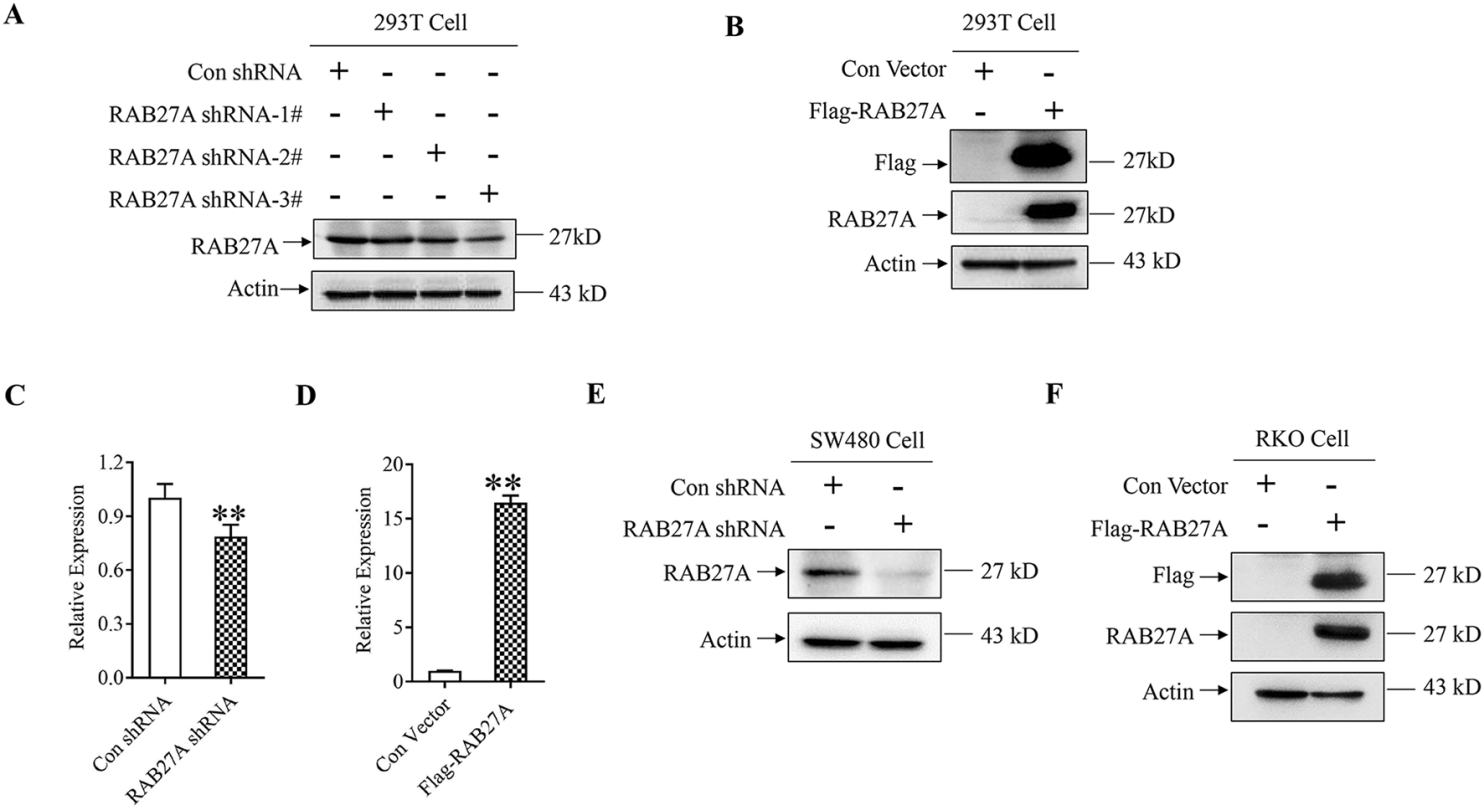
Identification of RAB27A knockdown and ectopic expression. (**A**) and (**B**). RAB27A knockdown and overexpression in 293 T cells. Cells were transfected with RAB27A shRNA and Flag-tag RAB27A plasmids, respectively. Then, western blotting was used to determine the expression level of RAB27A and Flag-tag RAB27A. Actin was used as a loading control. (**C**), (**D**), (**E**) and (**F**). Identification of RAB27A knockdown or overexpression in colon cancer cells. SW480 cells were transfected with RAB27A shRNA or Con shRNA plasmid, and RKO cells were transfected with Flag-tag RAB27A or the negative control plasmid. G418 (800 µg/ml) was used to select cell clones stably expressing neo gene. The mRNA level of RAB27A was measured by using real time PCR, and the protein level of RAB27A was determined by western blot. Actin was used as a loading control. The level of RAB27A mRNA was normalized by comparing mRNA level in all groups with that in the negative control groups. The relative expression level of RAB27A mRNA was shown as mean ± S.D. and was analyzed by two-tailed t-test. All the experiments were repeated three times independently, and the representative results were shown. **P < 0.01*.*

In our previous study, RAB27A expression in five CRC cell lines including HT-29, HCT116, SW480, RKO and LOVO was detected by western blot. The results indicated that RAB27A showed relatively high expression level in SW480 and low expression level in RKO (Fig. S1 and S2). Then, MTT and transwell assay were performed and the results indicated that the proliferation rate, as well as the migration and invasion ability, were higher in SW480 cells than that in RKO cells, which reveals that the expression level of RAB27A may positively correlate with the proliferation, migration and invasion ability of CRC cells (Fig. S3). Accordingly, SW480 and RKO were selected as cell models for RAB27A knockdown and overexpression, respectively. Meanwhile, RAB27A shRNA plasmid, Flag-tag RAB27A plasmid and the corresponding control plasmids were conducted to down-regulate or up-regulate RAB27A expression. As shown in Fig. 1A and S4, the protein level of RAB27A was significantly lower in 293 T cells transfected with RAB27A shRNA plasmids than that in the negative control group. In addition, transfection of Flag-tag RAB27A significantly up-regulated RAB27A expression in 293 T cells (Fig. 1B and S5). Therefore, RAB27A shRNA and Flag-tag RAB27A plasmids are efficient in mediating RAB27A knockdown and ectopic expression, respectively. Then, SW480 colon cancer cells were transfected with the selected RAB27A shRNA plasmid (3#), and RKO colon cancer cells were transfected with Flag-tag RAB27A plasmid. G418 sulfate (geneticin) was used to select cell clones that stably expressing neo gene. The results indicated that the expression level of RAB27A mRNA and protein were significantly lower in RAB27A knockdown SW480 cells than that in the negative control cells (Fig. 1C, E and S6). In contrast, RAB27A mRNA and protein level significantly increased in RKO cells stably transfected with Flag-tag RAB27A plasmid, compared with the negative control cells (Fig. 1D, F and S7).

In conclusion, we established the colon cancer cell lines stably down-regulating or up-regulating RAB27A expression by using RAB27A shRNA or Flag-tag RAB27A plasmids.

### RAB27A is involved in mediating the proliferation of colon cancer cells

**Figure 2:**
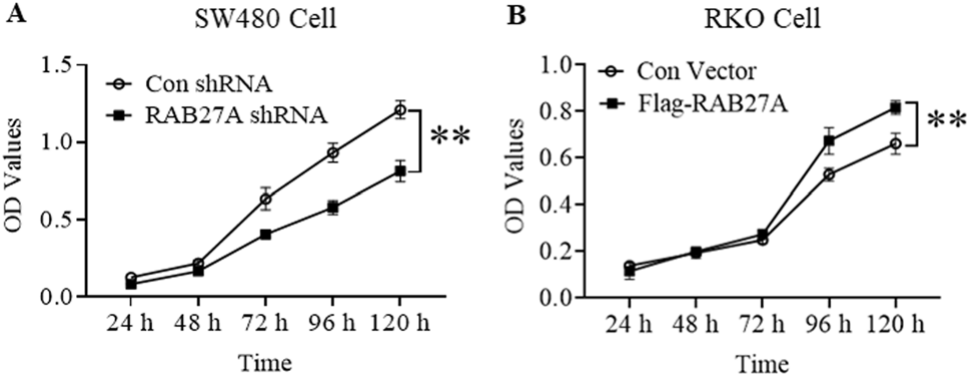
The effect of RAB27A on the proliferation of colon cancer cells. (**A**). RAB27A knockdown suppresses the proliferation of SW480 cells. The RAB27A knockdown and the negative control SW480 cells were seeded in 96-well plates, and cell proliferation was measured by using CCK-8 assay kit. (**B**). RAB27A overexpression promotes RKO cell proliferation. The RAB27A overexpression RKO cells and the negative control cells were seeded in 96-well plates, and cell proliferation was measured by using CCK-8 assay kit. The optical density (OD) value was shown as mean ± S.D. (n = 3), and analyzed by two-tailed *t*-test. **P < 0.01*.* All the experiments were repeated three times independently and the representative results were shown.

To explore the function of RAB27A on colon cancer proliferation, Cell Counting Kit-8 (CCK-8) was used to measure the growth of the established cell lines. As shown in Fig. 2A, the optical density (OD) value was significantly lower in RAB27A knockdown SW480 cells than that in the negative control cells since 48 h after cells were seeded, indicating that RAB27A knockdown inhibits SW480 cell growth. In contrast, the OD value in RKO cells stably expressing Flag-tag RAB27A was significantly higher than that in the negative control group since 72 h after cells were seeded (Fig. 2B), suggesting that RAB27A ectopic expression promotes RKO cell growth. Meanwhile, CCK-8 assay was also carried out in CRC cell line HCT116 and LOVO. As shown in Fig. S8, RAB27A was over-expressed in HCT116 and knocked-down in LOVO cells. And results of CCK-8 assay reveals that RAB27A over-expression promotes HCT116 cell proliferation, while RAB27A knockdown inhibits LOVO cell proliferation, which is in accordance with our results in SW480 and RKO.

**Figure 3:**
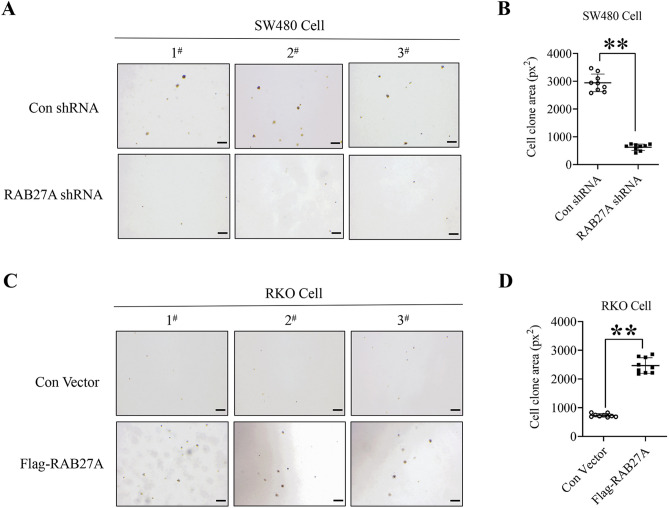
The effect of RAB27A on clone formation of colon cancer cells. (**A**) and (**B**). RAB27A knockdown inhibits clone formation of SW480 cells. RAB27A knockdown and the negative control SW480 cells were mixed with the melting agarose gel and plated in 6-well plates for 4 weeks, and cell clones were determined by microscopy (4 ×). (**C**) and (**D**). RAB27A overexpression promotes clone formation of RKO cells. The RAB27A overexpression and the negative control RKO cells were mixed with melting agarose gel and plated in 6-well plates for 4 weeks, and the cell clones were determined by microscopy (4 ×). At least three fields in each group were observed and the representative images were shown (scale bar = 200 µm). The area of cell clones was measured by using image J software, and was analyzed by two-tailed t-test. **P < 0.01. px^2^, the square of pixels. All the experiments were repeated three times independently and the representative results were shown.

In addition, we also determined the effect of RAB27A on cell clone formation by using soft agar colony formation assay. As shown in Fig. 3A and B, the area of cell clone in RAB27A knockdown SW480 cells was significantly smaller than that in the negative control cells, whereas cell colon area in RKO cells with stable Flag-tag RAB27A expression was significantly larger than that in the negative control group (Fig. 3C and D), indicating that RAB27A is of vital importance for clone formation of colon cancer cells.

Collectively, our data demonstrate that RAB27A plays a critical role in colon cancer cell growth.

### RAB27A is involved in mediating the migration and invasion of colon cancer cells

**Figure 4:**
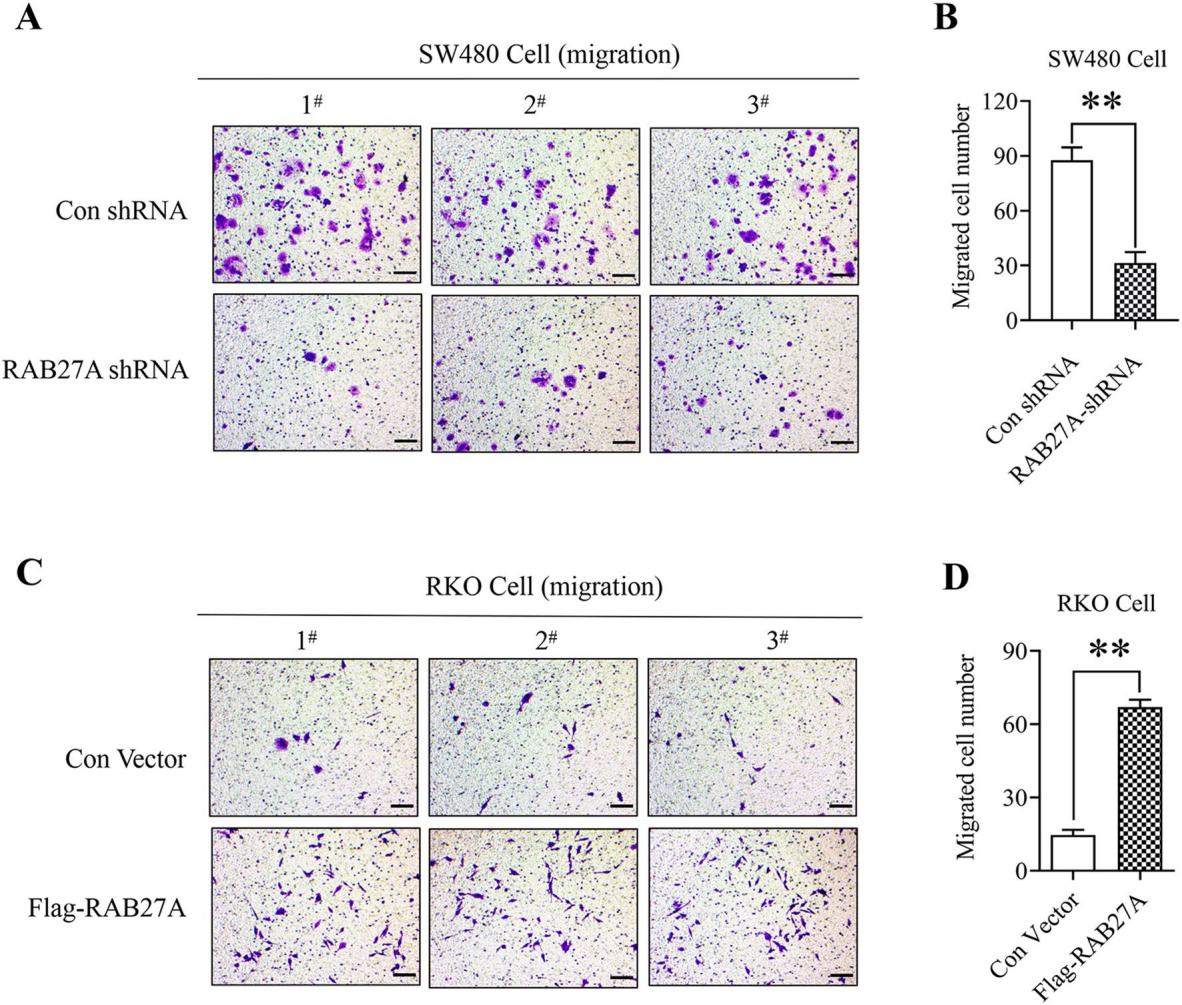
The effect of RAB27A on colon cancer cell migration. (**A**) and (**B**). RAB27A knockdown suppresses the migration of SW480 cells. RAB27A knockdown and the negative control SW480 cells were seeded in the upper well of the Transwell chamber for 48 h, and the migrated cells were stained with the crystal violet. (**C**) and (**D**). RAB27A overexpression facilitates RKO cell migration. RAB27A overexpression cells and the negative control cells were seeded in the upper well of the Transwell chamber for 48 h, and the migrated cells were stained with the crystal violet. Cell migration was determined by microscopy (200 ×). At least three fields in each group were observed, and the representative images were shown (scale bar = 100 µm). Migrated cell number in each field was shown as mean ± S.D. and was analyzed by two-tailed t-test. And the value of the vertical axes in this figure is migrated cell number per field of view. **P < 0.01. All the experiments were repeated three times independently and the representative results were shown.

**Figure 5:**
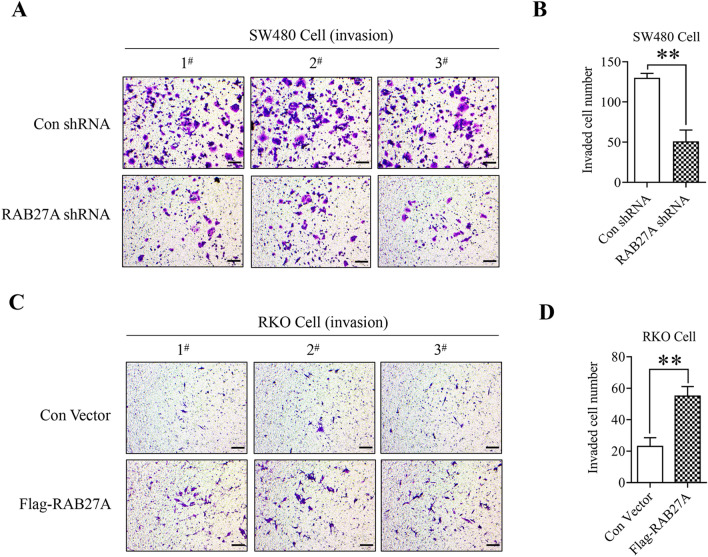
The effect of RAB27A on colon cancer cell invasion. (**A**) and (**B**). RAB27A knockdown suppresses SW480 cell invasion. RAB27A knockdown and the negative control SW480 cells were seeded in the upper well of the Transwell chamber pre-coated with Matrigel for 48 h, and the invaded cells were stained with the crystal violet. (**C**) and (**D**). RAB27A overexpression facilitates RKO cell invasion. RAB27A overexpression and the negative control RKO cells were seeded in the upper well of the Transwell chamber pre-coated with Matrigel for 48 h, and the invaded cells were stained with the crystal violet. Cell invasion was determined by microscopy (200 ×). At least three fields in each group were observed, and the representative images were shown (scale bar = 100 µm). Invaded cell number in each field was calculated, shown as mean ± S.D. and analyzed by two-tailed t-test. The value of the vertical axes in this figure is invaded cell number per field of view. **P < 0.01. All the experiments were repeated three times independently and the representative results were shown.

By using the stable cell lines of RAB27A knockdown and ectopic expression, we also explored the effect of RAB27A on cell migration and invasion by using the 24-well plate Transwell chambers with or without Matrigel. As shown in Fig. 4A and B, the migrated cell number in RAB27A knockdown SW480 cells was significantly lower than that in the negative control group, indicating that RAB27A plays a critical role in mediating the migration of SW480 colon cancer cells. Furthermore, we found that RAB27A ectopic expression promoted RKO cell migration (Fig. 4C and D), confirming the vital role of RAB27A in colon cancer cell migration. In addition, we also found that RAB27A knockdown inhibited SW480 cell invasion (Fig. 5A and B), whereas RAB27A overexpression facilitated RKO cell invasion (Fig. 5C and D), suggesting that RAB27A is of vital importance for colon cancer cell invasion. What is more, according to the results of cell proliferation analysis, Rab27a doesn’t affect the proliferation of SW480 or RKO cells within 48 h post cell passage. And the cell migration/invasion assay was completed within 48 h after cells were seeded. Therefore, in this study, the effect of Rab27a on cell proliferation has no impact on the results of cell migration/invasion assay.

In conclusion, RAB27A plays a key role in regulating the migration and invasion of colon cancer cells .

## Discussion

RAB27A was firstly identified as an oncogenic factor in melanoma, which has been found to promote melanoma cell proliferation via activating ERK signaling pathway^26^. Following studies demonstrate that RAB27A also promotes melanoma cell invasion and metastasis by facilitating the secretion of pro-invasive exosomes^18^. Moreover, RAB27A has been reported to promote the invasion and metastasis of breast cancer cells^20^. It is also demonstrated that RAB27A is highly up-regulated and acts as a valuable prognostic indicator in hepatocellular carcinoma^27^, glioma^23^, esophageal squamous cell cancer^28^ and clear cell renal cell carcinoma^29^. As for CRC, it has been reported that RAB27A promotes CRC tumor formation and growth via facilitating the secretion of VEGF and TGF-β in colon cancer cell line HT-29, which improves the self-renewal of colon cancer stem cells^24^. In HT-29 and HCT116, RAB27A knockdown suppresses exosome secretion, then inhibited exosome-induced proliferation and migration of endothelial cells^30^. In addition, ectopic expression of RAB27A abrogates the inhibitory effects of miR-582-5p on the proliferation and invasion of HCT116^31^. These results indicate that RAB27A acts as an oncogene in several types of cancer including CRC. In this study, we find that the proliferation, migration and invasion of colon cancer cells are significantly suppressed by RAB27A knockdown, but promoted by RAB27A ectopic expression. Therefore, our data demonstrate that RAB27A plays an oncogenic role in CRC progression, which is in accordance with previous studies. However, there are conflicting results on the function of RAB27A in CRC. It has been reported that RAB27A highly expresses in CRC tumor tissues compared with matched adjacent tissues, and high level of RAB27A protein positively correlates with lymph node metastasis and TNM stage but indicates favorable prognosis^32^. It is also reported that RAB27A is down-regulated in CRC primary tumor tissues compared with the adjacent tissues, and down-regulation of RAB27A is associated with poor differentiation, advanced TNM stage, distant metastasis, local recurrence and poor prognosis^25^. As mentioned above, both DWW et al. and Shi et al. reveal that high expression level of RAB27A correlates with favorable prognosis of CRC patients. However, the conclusions on the correlation between RAB27A expression and clinicopathological features are conflicting. This inconsistency may be attributed to the fact that the sample size of Shi’s research (112 CRC and 113 matched non-cancerous tissue specimens) is smaller than that of DWW’s research (388 CRC and 163 matched adjacent tissue specimens). Considering the heterogeneity of tumor tissues, the small sample size will affect the objectivity of the experiment and lead to greater statistical error, which may partially explain the discordance between the two studies and the relatively high *P*-value in Shi’s research. Therefore, in our view, the results of DWW’s research are more credible. That is to say, studies based on specimens derived from CRC patients demonstrate that RAB27A acts as a suppressor gene in CRC.

Interestingly, it seems that the function of RAB27A in tumor progression is inconsistent among different cancer types, even among different articles focusing on CRC. The conflicting role of the same gene in different cancer types is also observed in RAB25-related researches^33–35^. And this phenomenon may be attributed to two reasons. Firstly, function of RAB proteins is spatially and temporally regulated by binding to different effectors^36–38^. Secondly, the activities of exosomes in cancer progression are contradictory, which may partially explain the complicated role of RAB27A^32^. As for the conflicting conclusions on the role of RAB27A in CRC, we find that studies based on cell lines indicate the protumorigenic role of RAB27A^24,30,31^, while results of researches using specimens from CRC patients are the opposite^25,32^. Reasons that may explain this inconsistency are as follows. Firstly, the heterogeneity of tumor tissues and the subjectivity during the analysis of specimens may affect the objectivity of the data. Secondly, cell lines cultured in vitro cannot completely simulate the real physiological conditions. And more efforts are needed to explore the function of RAB27A in CRC progression.

Exosomes have been reported to be closely related to the progression of cancer, and RAB27A is an important regulator of exosome release^39^. It has been revealed that exosomes secreted from colon cancer cells promote the proliferation, invasion, and metastasis of colon cancer cells^40–42^. Moreover, knockdown of RAB27A in colon cancer cell line HCT116, SW480 and RKO inhibits exosome excretion, which significantly inhibits the promotion of CRC and endothelial cell migration induced by CRC-derived exosomes^43^. Similar results are also obtained in colon cancer cell line HT-29^30^. Therefore, we speculate that RAB27A may affect the malignant biological behavior of SW480 and RKO cells by regulating exosome secretion. Furthermore, MAPK/ERK signaling pathway plays a vital role in CRC progression and may be involved in RAB27A-mediated cancer progression^44–47^. It has been reported that PICALM promotes the proliferation and migration of HCT116 and RKO cells by activation of ERK/MAPK pathway^47^. In addition, hnRNPA2B1 significantly enhances cell proliferation and inhibits apoptosis in SW480 cells, meanwhile, the ERK/MAPK pathway is activated by up-regulation of hnRNPA2B1^46^. Similarly, in SW480 and HT-29 cells, overexpression of CLDN8 promotes cell proliferation and invasion via up-regulation of p-ERK and MMP9^45^. However, more experiments are still needed to confirm the conjectures in the future.

In summary, this is the first study to explore the exact function of RAB27A on CRC progression based on the stable cell lines of RAB27A knockdown and ectopic expression. We found that the proliferation, migration and invasion of colon cancer cells were significantly suppressed by RAB27A knockdown, but promoted by RAB27A ectopic expression. Therefore, RAB27A is of vital importance for colon cancer development, and may be a valuable prognostic indicator and potential therapeutic target for CRC.

## Materials and methods

### Cells and reagents

293 T cells, as well as colon cancer cell lines including SW480, RKO, HT-29, HCT116 and LOVO, were purchased from the Byotime Institute of Bio-technology (Jiangsu, China). Cells were cultured in Dulbecco's modified Eagle medium (DMEM; Gibco, Grand Island, NY, USA) containing 10% fetal bovine serum (FBS; Kangyuan Biology, China). The crystal violet was purchased from Sigma-Aldrich (St. Louis, MO, USA).

### Cell proliferation analysis

Enhanced Cell Counting Kit-8 (CCK-8) (Byotime, China) was used for cell viability test as per the manufacturer’s instructions. Cells were seeded in 96-well plates at the concentration of 3000 cells per well, and routinely cultured at 37 °C for 24 h. Subsequently, CCK-8 reagent (10 μl/well) was added into the culture medium at 24, 48, 72, 96 and 120 h after cells were seeded. At each time point, cells were incubated with the regent at 37 °C for 1–2 h, after which the OD value at 450 nm was measured by a microplate reader (Bio-Tek, Winooski, VT, USA). The color of the culture medium varies with the amount of formazan produced only by living cells, which is determined by detection of OD value at 450 nm. And the OD value shows linear positive correlation with the number of living cells.

### Soft agar colony formation

The low-melting-point agarose (Biowest, Spain) was prepared as 1.2 and 0.6% solution in ddH_2_O by using high pressure sterilization. The 0.6% agar/medium base layer was prepared by mixing 1.2% agarose solution with 2 × DMEM medium at 1:1 (volume), and then added to a 6-well cell culture plate to prevent cell attachment to the plastic base. Cells were collected by trypsinizatioin, counted and then suspended with DMEM medium at the density of 1 × 10^4^ cells/ml. The 0.3% agar upper layer was prepared by mixing 0.6% agarose with 2 × cell medium at 1:1 (volume). Subsequently, 1 ml upper agarose mixed with 100 µl single-cell suspension (~ 1,000 cells) were added to each well and solidified at room temperature. Cells were cultured at 37 °C in a humidified incubator containing 5% CO_2_ for 1 month, and cell clones were visualized by microscopy (4 ×). At least three random fields were selected and the cell clone area was calculated using image J software.

### Transwell migration and invasion assay

Cells transfected with either RAB27A knockdown or ectopic expression plasmid were collected and resuspended in DMEM. For cell migration assay, 4 × 10^4^ cells were seeded in the upper well of the Transwell chamber (Corning, NY, USA). And for cell invasion assay, 8 × 10^4^ cells were seeded in the upper well of the chamber pre-coated with Matrigel (Corning). Meanwhile, 600 µl DMEM containing 20% fetal bovine serum was added to the lower well of the chamber. The cells were routinely cultured for 48 h, after which cells on the upper-side of the membrane were cleaned out and cells on the underside were stained with 800 µl crystal violet for 30 min. At least three random fields were selected for cell counting.

### Western blotting

The cells were lysed with IP buffer (20 mM pH7.5 Tris, 150 mM NaCl, 1% Triton X-100) and protease inhibitors (0.1 mg/ml PMSF, 1 µg/ml Aprotinin, 1 µg/ml Leupeptin), and the protein concentration was determined by BCA assay (Beyotime, China). The proteins (30 µg per lane) were separated on a 12% polyacrylamide gel and transferred onto a PVDF membrane. Then the membrane was blocked with 5% non-fat milk at room temperature for 1 h and incubated with RAB27A monoclonal antibody (1:1000, Santa Cruz, CA, USA), ß-actin monoclonal antibody (1:5000, Proteintech, IL, USA) and flag polyclonal antibody (1:1000, MBL, Japan) at 4 °C overnight. After incubation with horseradish peroxidase-conjugated secondary antibody, the proteins were detected by ECL reaction system and gel-imaging system (Tanon, China).

### Real-Time PCR (qPCR)

Total RNA of the RAB27A interference and ectopic expression cells were extracted by RNA extraction Kit (BioTeKe, China), and then reverse-transcribed into cDNA by *EasyScript* First-Strand cDNA Synthesis SuperMix (TransGen Biotech, China) as per the manufacturer’s instructions. Real-time PCR was performed using BeyoFast™ SYBR Green qPCR Mix (Beyotime, China) and CFX96 Touch™ Real-Time PCR System (Bio-Rad, Hercules, CA, USA). The primer sequences were shown in Table 1. β-actin was used as control for normalization and 2^-△△CT^ method was used to calculate the relative expression of RAB27A.

**Table 1:** The primers used to amplify cDNA in Real-Time PCR.

Gene name	Forward	Reverse
Human RAB27A Human Actin	5′-ACAGCGTTCTTCAGAGATGCTATGG -3′ 5′- ACTCTTCCAGCCTTCCTTCC-3′	5′- TCTGCGAGTGCTATGGCTTCCT-3′ 5′- CGTACAGGTCTTTGCGGATG-3′

### RAB27A knockdown

The DNA fragments encoding shRNA targeting Rab27A were cloned into the GV102 vector. The constructed RAB27A interference plasmids and control plasmid were subsequently transfected into 293 T cells for 48 h. Total protein was extracted and western blotting was performed to select the plasmid with the highest interference efficiency, as described in “Western blotting” section. For stable transfection, SW480 cells were transfected with selected interference plasmid and control plasmid, then G418 (800 µg/ml) was used for resistance screening. The sequences of RAB27A shRNA and control shRNA were shown in Table 2.

**Table 2:** The sequences of RAB27A shRNA and control gene.

shRNA	RAB27A shRNA sequences
shRNA-1#	5′-GATCCCgcTGCCAATGGGACAAACATACTCGAGTATGTTTGTCCCATTGGCAGCTTTTTGGAT-3′ 3′-GGCGACGGTTACCCTGTTTGTATGAGCTCATACAAACAGGGTAACCGTcgAAAAACCTATCGA-5′
shRNA-2#	5′-GATCCCcaGGAGAGGTTTCGTAGCTTACTCGAGTAAGCTACGAAACCTCTCCTGTTTTTGGAT-3′ 3′-GGGTCCTCTCCAAAGCATCGAATGAGCTCATTCGATGCTTTGGAGAGGacAAAAACCTATCGA-5′
shRNA-3#	5′-GATCCCccTGTGCATTTGAATTGTATACTCGAGTATACAATTCAAATGCACAGGTTTTTGGAT-3′ 3′-GGGGACACGTAAAACTTAACATATGAGCTCATATGTTAAGTTTACGTGTccAAAAACCTATCGA-5′
Con shRNA	5′-GATCCCTTCTCCGAACGTGTCACGTCTCGAGACGTGACACGTTCGGAGAATTTTTGGAT-3′ 3′-GGAAGAGGCTTGCACAGTGCAGAGCTCTGCACTGTGCAAGCCTCTTAAAAACCTATCGA-5′

### RAB27A ectopic expression

Human RAB27A cDNA with Flag-tag sequence was cloned into the GV362 vector to express Flag-tag RAB27A and the plasmid was transfected into 293 T cells. And western blotting was performed to ensure the overexpression effect of the plasmid, as described in “Western blotting” section. To get the stable cell lines that express Flag-tag RAB27A, RKO cells were transfected with Flag-tag RAB27A plasmid, and G418 (800 µg/ml) was used to screen cells with high level of Flag-tag RAB27A expression.

### Statistical analysis

GraphPad prism 7 software was used to analyze the data and construct statistical graphs. If variance was homogeneous, statistical significance was analyzed by performing two-tailed Student's *t*-test, otherwise, the *t*’-test was used for comparison between two groups. *P* < 0.01 was defined as significantly different. All experiments were repeated three times independently, and the data were expressed as mean ± S.D. from representative experiments.

## Supplementary Information

Supplementary Information.
