# Author Correction: RAB27A promotes the proliferation and invasion of colorectal cancer cells

**DOI:** 10.1038/s41598-023-27867-y

**Published:** 2023-01-13

**Authors:** Qingyan Li, Huixia Zhao, Weiwei Dong, Na Guan, Yanyan Hu, Zhiyan Zeng, He Zhang, Fengyun Zhang, Qiuwen Li, Jingwen Yang, Wenhua Xiao

**Affiliations:** 1 Graduate School of Jinzhou Medical University, Liaoning, 121001 China; 2 Senior Department of Oncology, the Fifth Medical Center of PLA General Hospital, Beijing, 100071 China; 3 Department of Oncology, 4th Medical Center of PLA General Hospital, Beijing, 100048 China; 4 Department of Oncology, Suining Central Hospital, Sichuan, 629300 China

Correction to: *Scientific Reports* 10.1038/s41598-022-23696-7, published online 12 November 2022

The original version of this Article contained errors in Figure 4 where the figures “1^#^” and “3^#^” in “Con shRNA” in panel (a) were incorrect. The original Figure 4 and accompanying legend appear below.

**Figure 4:**
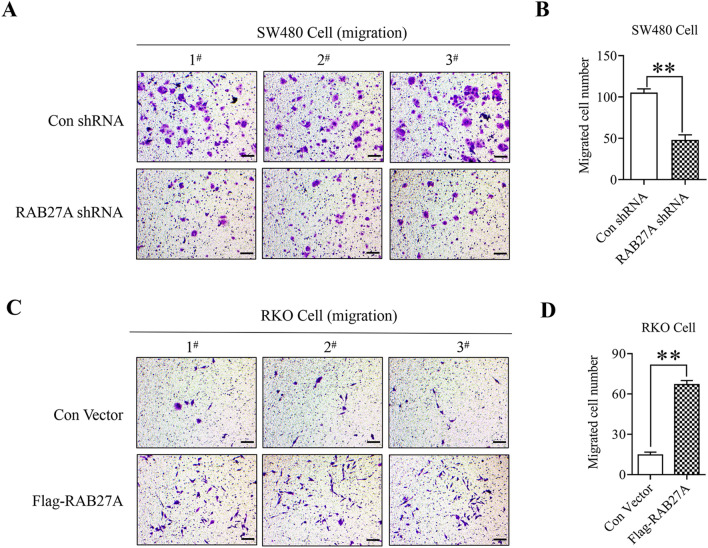
The effect of RAB27A on colon cancer cell migration. (**A,B**) RAB27A knockdown suppresses the migration of SW480 cells. RAB27A knockdown and the negative control SW480 cells were seeded in the upper well of the Transwell chamber for 48 h, and the migrated cells were stained with the crystal violet. (**C,D**) RAB27A overexpression facilitates RKO cell migration. RAB27A overexpression cells and the negative control cells were seeded in the upper well of the Transwell chamber for 48 h, and the migrated cells were stained with the crystal violet. Cell migration was determined by microscopy (200×). At least three fields in each group were observed, and the representative images were shown (scale bar = 100 µm). Migrated cell number in each field was shown as mean ± S.D. and was analyzed by two-tailed t-test. And the value of the vertical axes in this figure is migrated cell number per field of view. **P < 0.01. All the experiments were repeated three times independently and the representative results were shown.

The original Article has been corrected.

