# Supplementary material for: RAB27A promotes the proliferation and invasion of colorectal cancer cells
Source: Sci Rep. 2022 Nov 12;12:19359. doi: 10.1038/s41598-022-23696-7 (PMC9653419; doi:10.1038/s41598-022-23696-7)
Supplement: Supplementary file 1 — Supplementary Information. [file 41598_2022_23696_MOESM1_ESM.pdf]

Supplementary Information

Title: RAB27A promotes the proliferation and invasion of colorectal cancer cells

Author information

Affiliations

Graduate School of Jinzhou Medical University, Jinzhou, 121001, Liaoning, China.

Qingyan Li, Na Guan & Wenhua Xiao

Senior Department of Oncology, the Fifth Medical Center of PLA General Hospital, Beijing, 100071, China.

Qingyan Li, Weiwei Dong, Na Guan, Yanyan Hu, Qiuwen Li, Jingwen Yang &Wenhua Xiao

Department of Oncology, 4th Medical Center of PLA General Hospital, Beijing, 100048, China.

Huixia Zhao, Zhiyan Zeng, He Zhang & Fengyun Zhang

Department of Oncology, Suining Central Hospital, Suining, 629300, Sichuan, China.

Qingyan Li

***Correspondences and requests for materials should be addressed to:**

Wenhua Xiao. Senior Department of Oncology, the Fifth Medical Center of PLA General Hospital, #8 East Street, Fengtai District, Beijing, 100071, China. Tel.: 8613681532672. E-mail: w_hxiao@hotmail.com

Jingwen Yang. Senior Department of Oncology, the Fifth Medical Center of PLA General Hospital, #8 East Street, Fengtai District, Beijing, 100071, China. Tel.: 8613488887450. E-mail: 8537505@sina.com

Supporting figures

Figure S1-S2. (P3-P4)

The expression of RAB27A in different CRC cell lines.

Figure S3. (P5)

Detection of proliferation, migration and invasion ability in SW480 and RKO cells.

Figure S4-S7. (P6-P9)

Full-length western blot images of Figure 1A, 1B, 1E and 1F.

Figure S8. (P10)

The effect of RAB27A on the proliferation of colon cancer cell line HCT116 and LOVO.

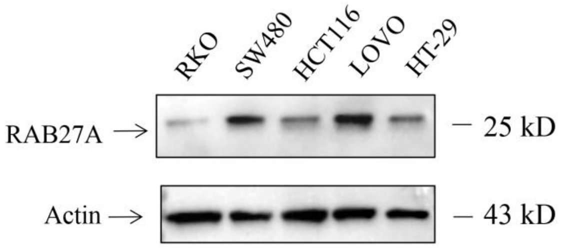

Figure S1. The expression of RAB27A in different CRC cell lines. Western blotting was performed to detect the expression of RAB27A in different CRC cell lines including RKO, SW480, HCT116, LOVO and HT-29. Actin was used as a loading control.

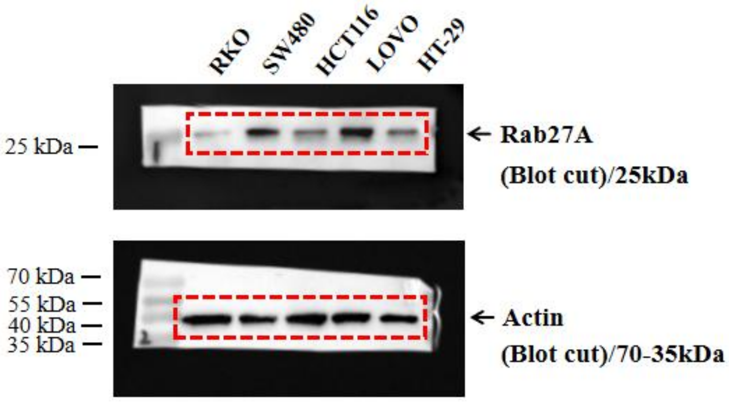

Figure S2. Full-length western blot images of Figure S1. The expression of RAB27A in different CRC cell lines. Western blotting was performed to detect the expression of RAB27A in different CRC cell lines including RKO, SW480, HCT116, LOVO and HT-29. Actin was used as a loading control.

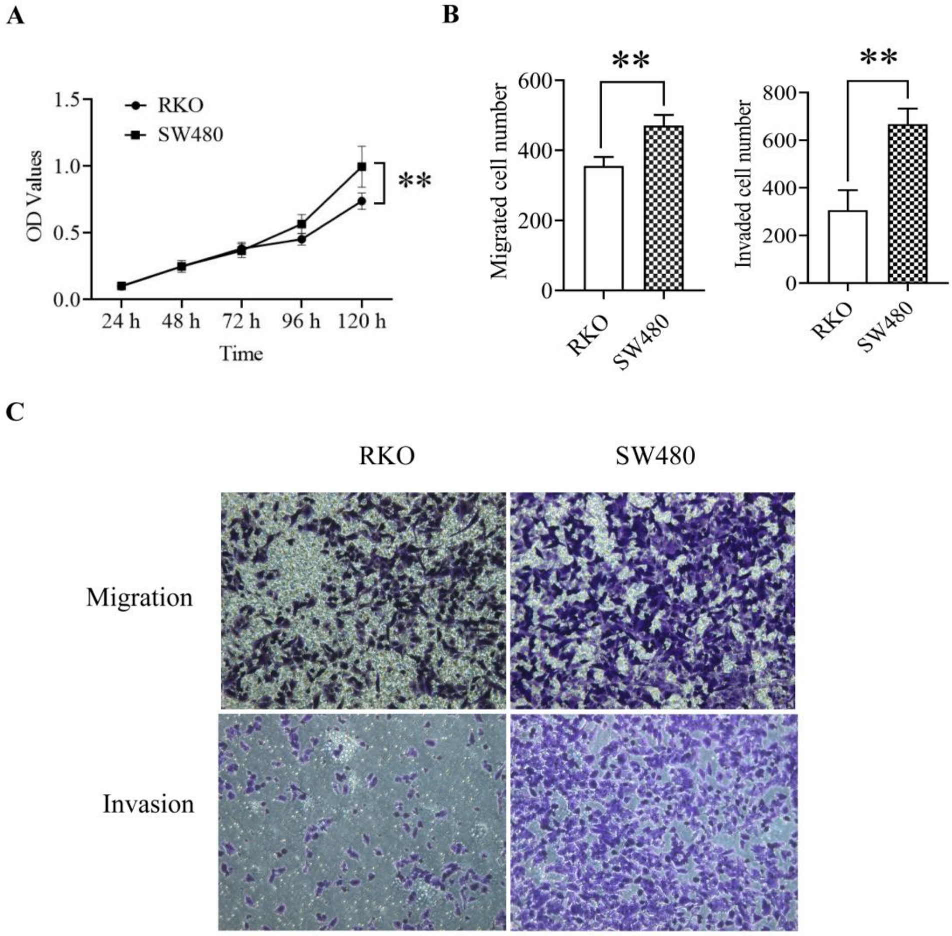

Figure S3. Detection of proliferation, migration and invasion ability in SW480 and RKO cells. (A). MTT was performed and the results indicated that the proliferation rate of SW480 was higher than that of RKO. (B) and (C). Transwell assay was carried out and the results showed that the number of migrated/invaded cells in SW480 was higher than that in RKO. Cell migration/invasion was determined by microscopy (200×). At least three fields in each group were observed, and the representative images were shown. Migrated/invaded cell number in each field was shown as mean ± S.D. and was analyzed by two-tailed t-test. And the value of the vertical axes in this figure is migrated/invaded cell number per field of view. ***P*<0.01.

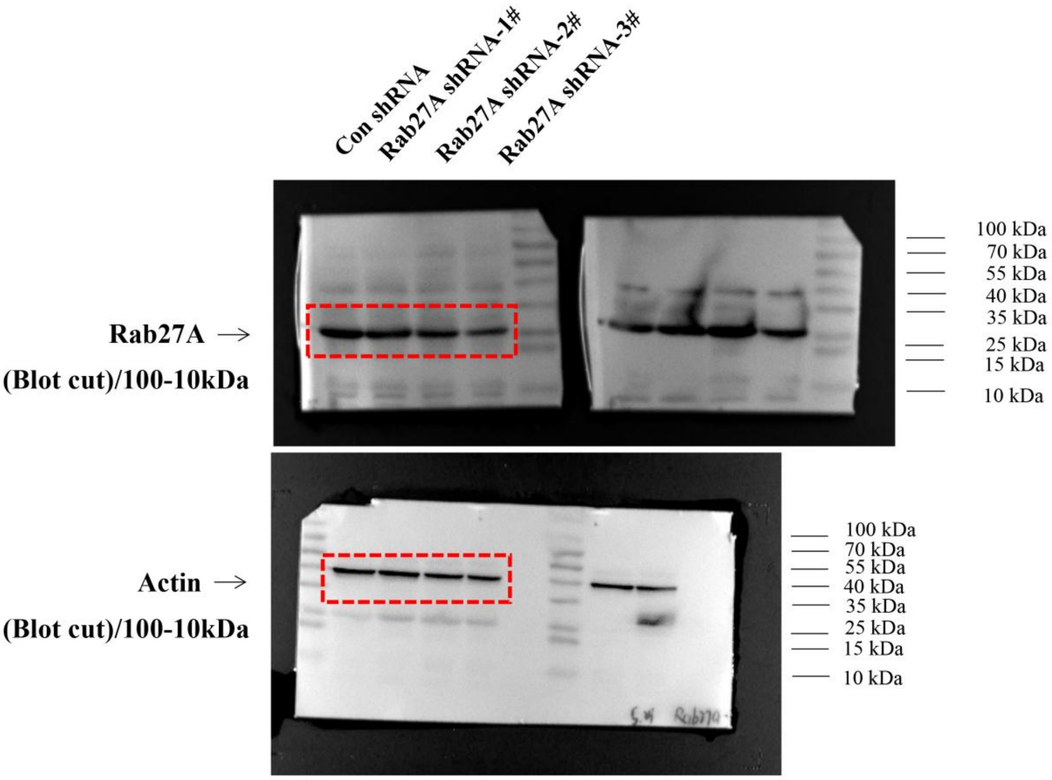

Figure S4. Full-length western blot images of Figure 1A. 293T cells were transfected with RAB27A shRNA or control plasmid. And western blotting was used to determine the expression level of RAB27A. Actin was used as a loading control.

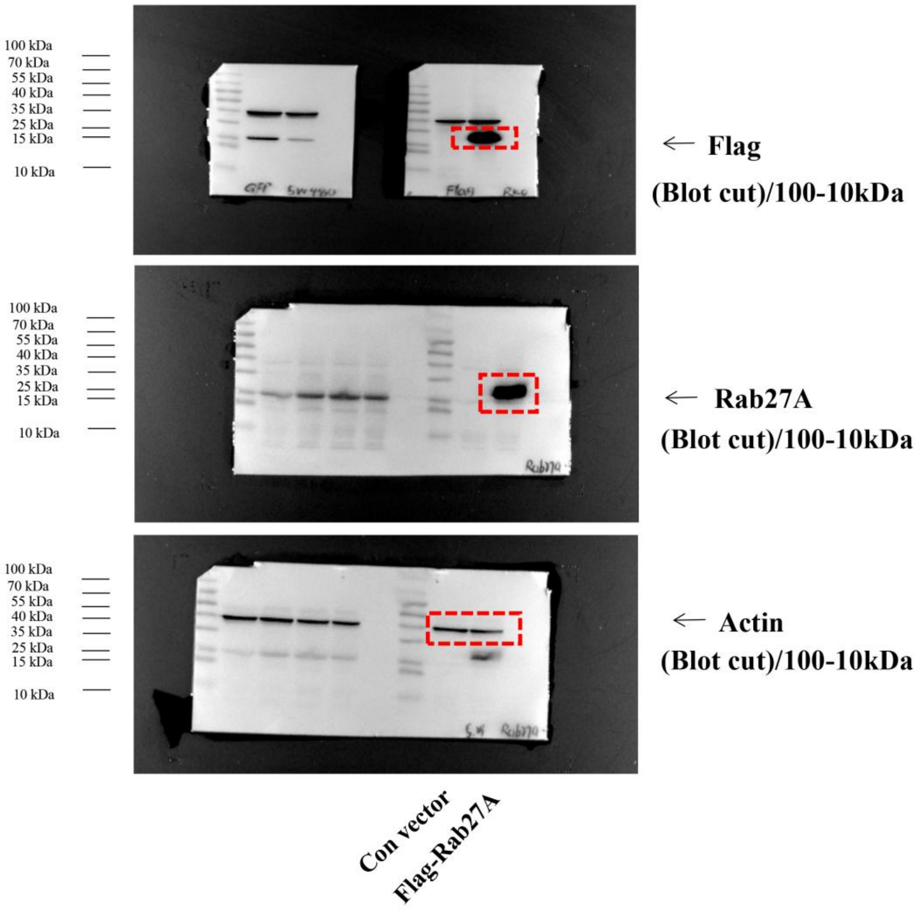

Figure S5. Full-length western blot images of Figure 1B. 293T cells were transfected with Flag-tag RAB27A or control plasmid. And western blotting was used to determine the expression level of RAB27A and Flag-tag RAB27A. Actin was used as a loading control.

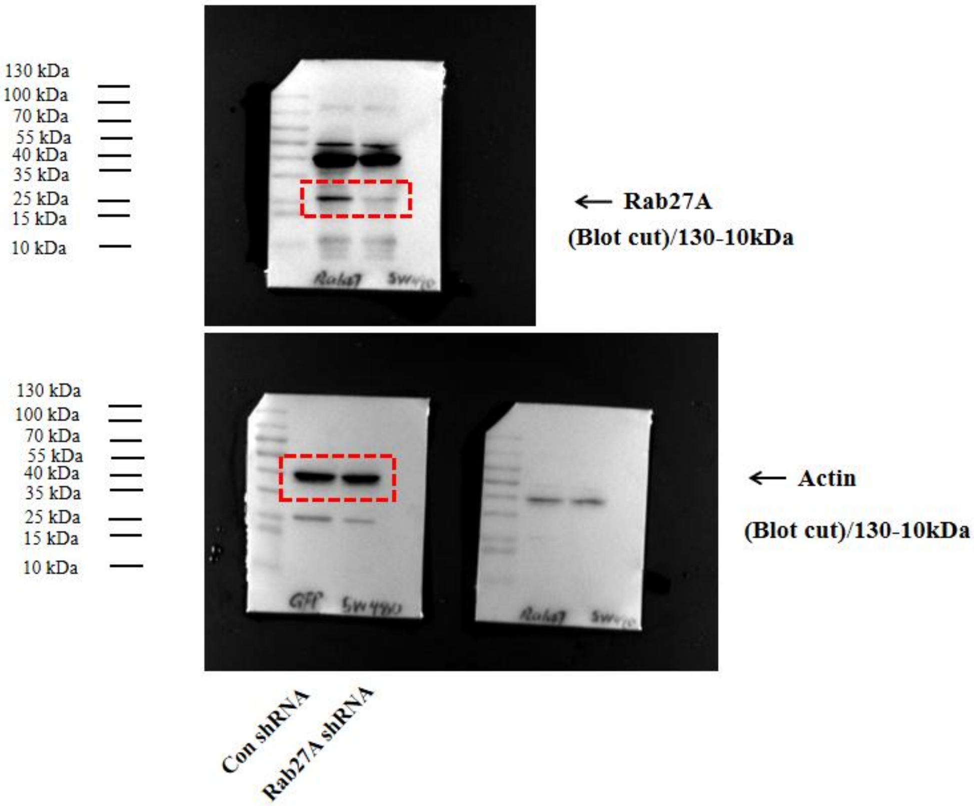

Figure S6. Full-length western blot images of Figure 1E. SW480 cells were transfected with RAB27A shRNA or control plasmid. And western blotting was used to determine the expression level of RAB27A. Actin was used as a loading control.

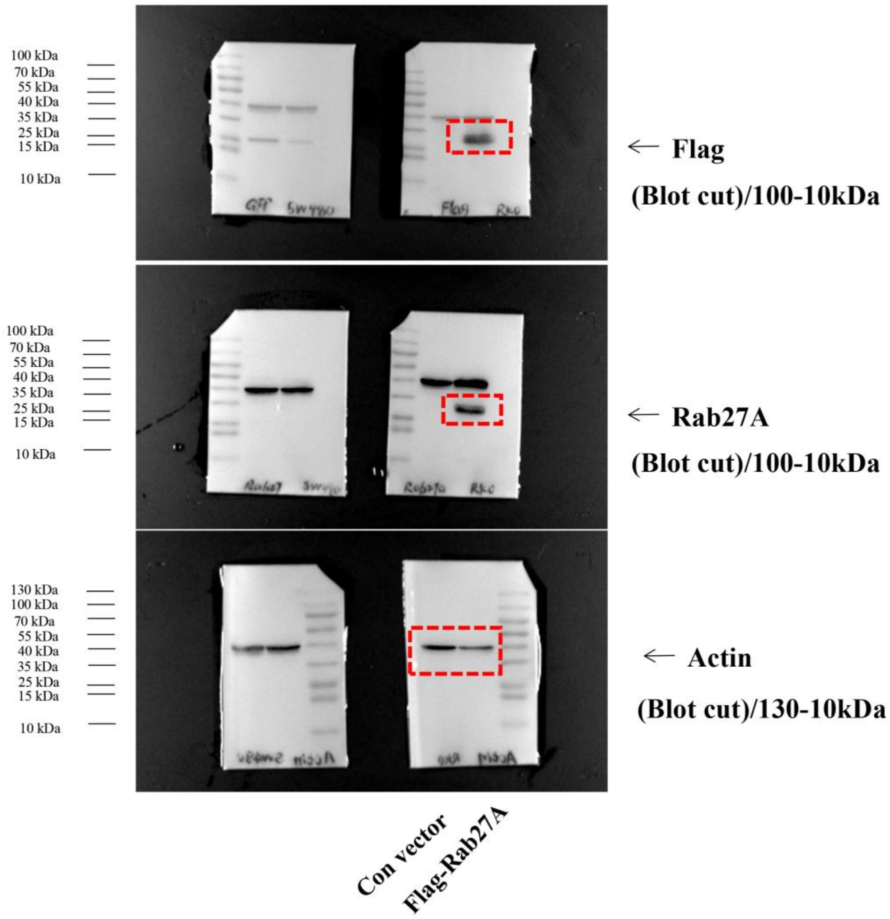

Figure S7. Full-length western blot images of Figure 1F. RKO cells were transfected with Flag-tag RAB27A or control plasmid. And western blotting was used to determine the expression level of RAB27A and Flag-tag RAB27A. Actin was used as a loading control.

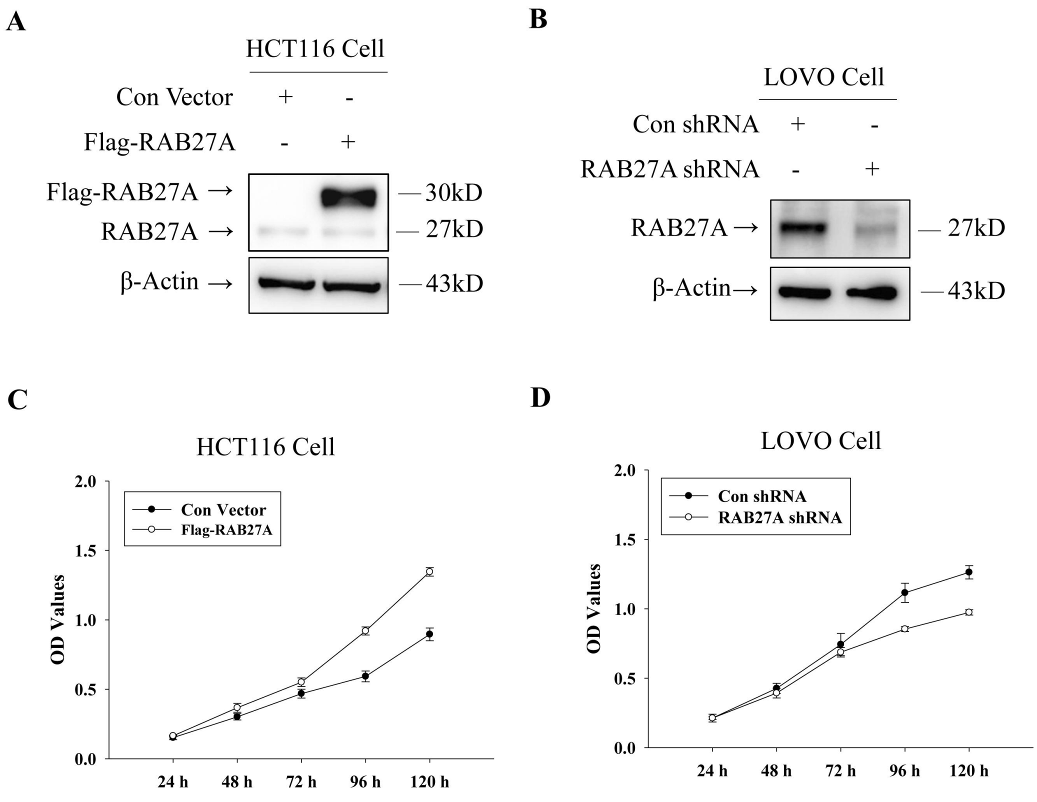

Figure S8. The effect of RAB27A on the proliferation of colon cancer cell line HCT116 and LOVO. (A). Western blot was performed and the results indicate that RAB27A is over-expressed in HCT116 cells. (B). Western blot was performed and the results indicate that RAB27A is knocked-down in LOVO cells. (C). CCK-8 assay was performed and the results reveal that RAB27A over-expression promotes HCT116 cell proliferation. (D). CCK-8 assay was performed and the results reveal that RAB27A knockdown suppress the proliferation of LOVO cells.
